# Contrast Induced Nephropathy with Intravenous Iodinated Contrast Media in Routine Diagnostic Imaging: An Initial Experience in a Tertiary Care Hospital

**DOI:** 10.1155/2016/8792984

**Published:** 2016-03-16

**Authors:** Shuchi Bhatt, Nipun Rajpal, Vineeta Rathi, Rajneesh Avasthi

**Affiliations:** ^1^Department of Radiodiagnosis, University College of Medical Sciences and GTB Hospital, Dilshad Garden, Delhi 110095, India; ^2^Department of Medicine, University College of Medical Sciences and GTB Hospital, Dilshad Garden, Delhi 110095, India

## Abstract

*Background*. Contrast induced nephropathy (CIN) is common cause of hospital acquired renal failure, defined as iatrogenic deterioration of renal function following intravascular contrast administration in the absence of another nephrotoxic event.* Objectives*. Objectives were to calculate incidence of CIN with routine IV contrast usage and to identify its risk factors.* Materials and Methods*. Study was conducted on 250 patients (having eGFR ≥ 45 mL/min/1.73 m^2^) receiving intravenous contrast. Various clinical risk factors and details of contrast media were recorded. Patients showing 25% increase in postprocedural serum creatinine value or an absolute increase of 0.5 mg/dL (44.2 mmol/L) were diagnosed as having CIN.* Results and Conclusions*. Postprocedural serum creatinine showed significant increase from baseline levels. 25 patients (10%) developed CIN. CIN was transient in 21 (84%) patients developing CIN. One patient (4%) developed renal failure and another died due to unknown cause. Dehydration, preexisting renal disease, cardiac failure, previous contrast administration, and volume of contrast had significant correlation with development of CIN (*p* < 0.05); whereas demographic variables, baseline serum creatinine/eGFR, previous renal surgery, diabetes mellitus, hypertension, nephrotoxic drug intake, abnormal routine hematology, and contrast characteristics had no correlation with CIN. CIN is a matter of concern even in routine imaging requiring intravenous contrast media, in our set-up.

## 1. Introduction

Contrast induced nephropathy (CIN) is iatrogenic deterioration of renal function following intravascular contrast media (CM) administration in the absence of another nephrotoxic event. It is considered to be the third most common cause of hospital acquired acute renal failure [1]. Recently there also has been a controversy over the term CIN, and the Acute Kidney Injury Network (AKIN) has adopted “Acute Kidney Injury (AKI)” as a synonym of CIN when AKI occurs due to contrast administration [2]. However, the use of the term CIN is still popular among the radiologists and cardiologists dealing with contrast.

CIN in most cases goes undetected especially if the investigation is done in an outpatient setting. Most of the episodes of CIN are transient and resolution occurs within 1 to 3 weeks, but in a few cases permanent impairment of renal function may occur, reflected as an increase in serum creatinine [3]. Though serum creatinine is a late and an insensitive marker, it remains the cornerstone for diagnosing CIN due to its low cost and ease of estimation.

CIN is associated with both short and long term adverse outcomes and therefore the recognition of this condition is necessary [3].

Till date no universally accepted definition of CIN exists and therefore a variable incidence has been reported in the literature ranging from 1.3 to 14.5% depending upon the criteria used [4]. The most accepted definition is that of the European Society of Urogenital Radiology (ESUR) which defines CIN as “an increase in serum creatinine by >25% or an absolute increase of 44.2 mmol/L [0.5 mg/dL] within 3 days after intravascular administration of contrast medium, without an alternative etiology” [5]. The magnitude of CIN risk has been evaluated more for intra-arterial contrast especially in patients undergoing cardiac angiography where the reported incidence of 3–16% is relatively higher [3, 6, 7].

In the recent times, with the development of multidetector CT technology allowing enhanced clinical applications of CT especially CT angiographies, the intravenous use of CM has increased many folds. This voluminous increase in the use of contrast has generated an interest among the investigators to assess its safe intravenous administration with respect to the renal status. CIN due to intravenous use of contrast has not been adequately evaluated and the existing studies too have contradictory results. An incidence of 11% had been reported with intravenous (IV) contrast in patients undergoing emergency Contrast Enhanced Computed Tomography (CECT) examination in a recent study [8]. However, in another study the occurrence of CIN with IV contrast media was only considered to be coincidental [9].

It is extremely important to study the risk factors in the target population and thus identify the high risk patients. Already established patient factors responsible for causing CIN are preexisting renal insufficiency, diabetes mellitus, dehydration before and after the contrast procedure, congestive cardiac failure, and advanced age. Certain contrast related risk factors like the volume and type of contrast administered have also been identified in the development of CIN [10–14].

There is a compelling need for the clinicians and the radiologists to recognize this definite risk associated with the intravenous contrast usage in the radiology department. This study was conducted with the aim to determine the incidence of contrast induced nephropathy (CIN) in low risk patients undergoing routine diagnostic imaging like Intravenous Urography (IVU) or Contrast Enhanced Computed Tomography (CECT) examination with intravenous administration of iodinated contrast media and to identify the patient and contrast related factors responsible for CIN. The secondary objectives of the study were to compare the incidence of contrast induced nephropathy between ionic and nonionic contrast media and to suggest guidelines for the routine working of the department to achieve safe intravenous contrast administration.

## 2. Materials and Methods

The study was a cross-sectional study conducted in the Department of Radiodiagnosis, University College of Medical Sciences and Guru Tegh Bahadur Hospital, Delhi, between November 2012 and April 2014.

After taking clearance from the institutional ethical committee, the study was carried along the following lines.

Sample size considering the incidence of CIN to be 11% as reported in a prospective study on CECT patients [8] and allowing an absolute error of 4% (11 ± 4%), a minimum sample size of 234 patients was required to achieve a confidence interval of 95% in the study.

We enrolled 390 unbiased samples of adult patients coming for Intravenous Urography (IVU) or Contrast Enhanced Computed Tomography (CECT) examination and requiring intravenous administration of contrast media. A written informed consent was obtained from the patients. The demographic details, suspected clinical diagnosis, and preliminary investigations were recorded on predesigned proforma. History of allergy to contrast media/any other drug was elicited and recorded. Alternative investigation was recommended to patients allergic to contrast to answer the clinical question. In case the contrast study was deemed necessary by the clinician, the contrast investigations were performed using low osmolal nonionic contrast media after premedication with steroids as per guidelines. These patients were excluded from the study population. Serum creatinine was repeated if the preprocedural report was more than 1 week old from the date of investigation.

Estimated Glomerular Filtration Rate (eGFR) of the patient was calculated by the resident (NR) using the Modification of Diet in Renal Disease (MDRD) equation [15]:

(1) eGFRmL/min/1.73 m2=186×serum creatinine1.154×Age−0.203×0.742  if female×1.210  if African American.

Patients requiring preventive hydration therapy having eGFR < 45 mL/min/1.73 m^2^ [16] were excluded from the study. Review for the need of the investigation was done in consultation with the concerned physician and the investigation performed in 14 such patients using preventive strategies as per Consensus Guidelines for the Prevention of Contrast Induced Nephropathy by Canadian Association of Radiologists [15] and were recorded separately.

Patients who refused to give consent were excluded. Patients who did not receive intravenous (IV) contrast or patients having an eGFR of less than 45 mL/min/1.73 m^2^ were excluded. Patients whose postprocedural serum creatinine was not available were not included in the study. Therefore, the final study sample consisted of 250 patients comprised of 64.1% of the total enrolled patients.

Patients were interrogated regarding the presence of any known risk factor according to a predesigned questionnaire and their answers were recorded. Appropriate laboratory investigations were advised when history suggestive of one or more risk factors was present. The available clinical records of the patients were checked for the presence of any defined risk factor. Appropriate routine instructions were given to the patients for the prospective intravenous contrast investigation.

The following risk factors were identified representing a blend of available literature.

*Dehydration.* Patients having recent history of prolonged diarrhoea or vomiting or having limited oral intake in recent past with history of recent weight loss and lethargic look were clinically examined and labelled as dehydrated if dry mucous membranes and abnormal skin turgor were present.

Preexisting renal disease on basis of structural (e.g., single kidney and renal cell carcinoma) and functional abnormality (e.g., raised previous serum creatinine) of kidneys on previous investigations was identified as separate risk factor. Patients having only calculus or mild hydronephrosis but normal function of kidney were not included.

*Previous Renal Surgery.* History of previous renal surgery like nephrectomy, pyelolithotomy, and so forth was also identified as a separate risk factor.

*Diabetes Mellitus.* Patient who was a known case of diabetes mellitus on antidiabetic treatment (on oral hypoglycemic drugs or on insulin) or had recent fasting blood glucose >126 mg/dL was identified as a separate risk factor [17].

*Hypertension.* Patient is a known case of hypertension on antihypertensive drugs or has blood pressure >140/90 mm of Hg [18].

*Cardiac Failure.* Patients have a past or present documented history of cardiac failure.

*Nephrotoxic Drug Intake.* Patients use nephrotoxic drugs like NSAIDs, beta blockers, aminoglycosides, or amphotericin B.

*Previous Contrast Use.* Patients who had undergone previous contrast study were considered as a separate risk factor. Patients having history of intravascular iodinated contrast study within two weeks of the contrast investigation were included in this study.

*Abnormal Routine Blood Investigations.* Abnormal routine blood investigations were considered as separate risk factor which included anaemic patients (with haemoglobin level of less than 13 g/dL and less than 12 g/dL in women) [19] or laboratory findings suggestive of infection like leukocytosis (value greater than 11000/*μ*L) or patients with elevated CRP [20].

Patients with history of gout, multiple myeloma, or hyperthyroidism were also considered to be risk factors. However, no such patient was present in our study.

The type and amount of contrast media given to the patient were decided as per standard protocol being followed routinely in the department.

After completion of the investigation (IVU or CECT), volume, and type of contrast media, the total iodine content or any reaction if occurred was recorded.

Contrast was subdivided on the basis of ionicity into ionic and nonionic; osmolarity into high osmolal contrast media (HOCM) and low osmolal contrast media (LOCM); structure into monomer and dimer. The Following contrasts were used in the study population: iohexol (nonionic, monomer, and LOCM); sodium meglumine diatrizoate (ionic, HOCM, and monomer); iopamidol (nonionic, monomer, and LOCM); and ioxaglate (Ionic, LOCM, and dimer).

Repeated serum creatinine estimation was done 48–72 hrs after contrast (IVP or CECT) investigation.

After the contrast administration, patients showing an increase in serum creatinine by 25% or an absolute increase of 0.5 mg/dL from preprocedural level were diagnosed as having contrast induced nephropathy [5].

In patients who were diagnosed as CIN, serum creatinine was repeated weekly till it reached the pre procedural values.

All patients with CIN were followed 4–6 weeks and were watched for features of renal deterioration like oliguria, symptoms related to pulmonary edema or any metabolic disturbances, and were recorded separately.

## 3. Outcome Measures

The primary end point of this study was CIN defined as an increase in the postprocedural serum creatinine value by 25% from the baseline or an absolute increase of 0.5 mg/dL (44.2 mmol/L) within three days to intravascular contrast administration [5]. This definition of CIN was chosen over the recently proposed relatively lower threshold of 0.3 mg/dL for defining AKI (due to contrast administration) by the AKIN [2]. The former is more specific and less likely to yield a false-positive result from cumulative biologic and assay variability and remains a more commonly used definition in current medical practice [21–23]. Thus, this has been used to define CIN in the present study.

### 3.1. Statistical Analysis

The collected data was entered into a spreadsheet format using Microsoft Office Excel. Data was processed using statistical software SPSS version 17.1.

The incidence of CIN was calculated in the study population and separately for nonionic and ionic contrast media groups. The calculated incidence was compared with previously reported incidence using tests of proportions, for which *p* < 0.05 was considered statistically significant.

The baseline preprocedural and postprocedural serum creatinine values were compared for any difference and evaluated whether the difference was significant using paired *t*-test.

For evaluation of eGFR, because of nonnormal distribution, a nonparametric method (Mann-Whitney *U* test) was used.

Risk factors responsible for CIN were identified by estimating their distribution in the CIN and the non-CIN groups. Continuous variables including age, weight, volume of contrast, and total iodine given to patient were presented as mean ± SD and compared using Student's *t*-test.

Categorical variables including dehydration, preexisting renal disease, history of renal surgery, diabetes mellitus, hypertension, nephrotoxic drug Intake, heart failure, previous contrast use, and the osmolarity of the contrast media were presented as counts and percentages and compared with Fisher's exact test. Categorical variables including sex, abnormal routine hematologic investigations, administered contrast volume subgroups, and the ionicity of the contrast were compared using chi-square tests.

Results of this model were presented as relative risk (RR) with 95% confidence intervals using univariate analysis.

## 4. Results

**Figure 1:**
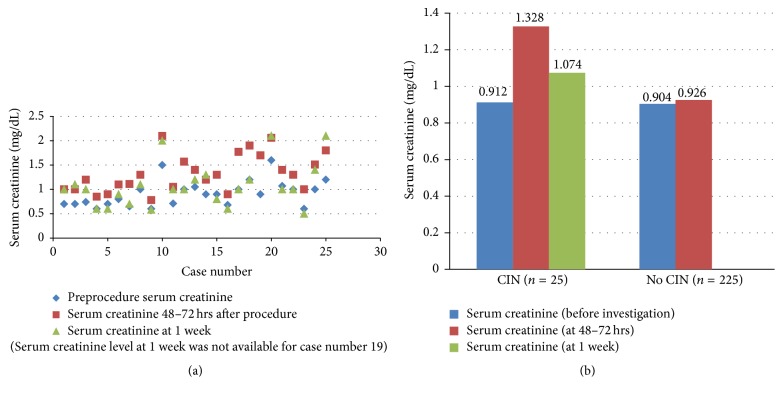
(a) Scatter diagram depicting serum creatinine levels at different time intervals in “CIN” patients. (b) represents the mean serum creatinine values in the pre- and post-IV iodinated contrast investigation in the CIN and the No CIN groups.

All 250 patients in our study were adults with age ranging from 18 to 86 years with the mean age of 41.41 ± 16.63 years. The sample size included 147 (58.8%) male and 103 (41.2%) female patients; 201 (80.4%) underwent contrast enhanced CT examination while in the rest 49 (19.6%) patients Intravenous Urography (IVU) was done. In these 250 patients, mean preprocedural serum creatinine was 0.905 ± 0.248 mg/dL which increased to 0.966 ± 0.300 mg/dL after 48–72 hours of the contrast investigation. This increase in serum creatinine was found to be statistically significant using paired *t*-test with *p* value < 0.001 (Figure 1).

A total of 25 (10%) of the 250 patients in sample group developed CIN as per the definition of European Society of Urogenital Radiology. Therefore, the incidence of CIN found to be 10% (95% CI 6.8% to 14.4%) in our study.

Out of a total of 187 patients who were given nonionic contrast medium, 20 satisfied the criteria of CIN; and 5 out of the total of 58 patients who were given ionic contrast medium developed CIN. The incidence of CIN is 10.7% in the nonionic group and 7.9% in the ionic group.

The patient sample was divided into 2 groups “CIN group” and “No CIN group,” the distribution of the various risk factors studied in these two groups to calculate their association with CIN.

Following univariate analysis, analysis of different features revealed the CIN and No CIN groups to be homogeneous for the demographic characteristics including age, sex, and weight with *p* > 0.05 (Table 1).

Both the groups were not significantly different with respect to the age, sex, or weight of the patients and therefore these demographic factors were not found to affect the incidence of the occurrence of CIN with the IV contrast media.

**Figure 2:**
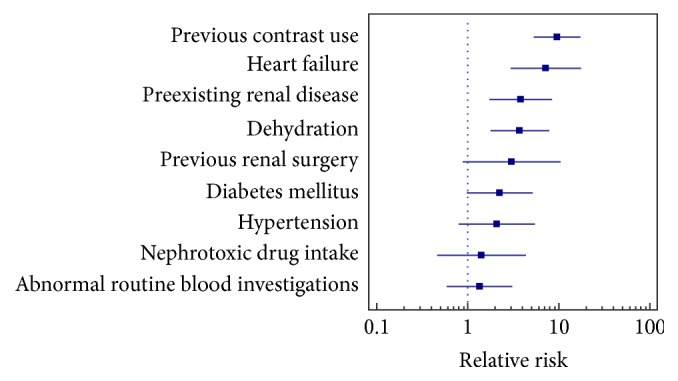
Forest plot depicting relative risk of clinical risk factors (result of univariate analysis).

Regarding clinical risk factors (Table 2), it was found that the CIN group had significantly higher proportion of patients with dehydration, preexisting renal disease, previous contrast administration, and cardiac failure (*p* < 0.05) with high relative risks. Previous renal surgery, diabetes mellitus, and hypertension were not statistically significantly different in both groups with *p* > 0.05 with relative risks of 3.02 (95% CI 0.88–10.37), 2.23 (95% CI 0.97–5.15), and 2.08 (95% CI 1.79–5.49), respectively (Figure 2).

Patients with history of nephrotoxic drug intake and abnormal routine blood investigations were found to be homogenously distributed in both CIN and No CIN groups with low relative risks.

### 4.1. Contrast Related Risk Factors

“CIN” and “No CIN” groups were homogenous in terms of ionicity, osmolarity, and molecular structure of contrast (*p* > 0.05) while mean volume of contrast administered was significantly higher in CIN group (*p* < 0.05). Mean total iodine given to patients in “CIN” group was higher which was the limit of significance (*p* = 0.05) (Table 3).

In the CIN group, renal function returned to normal within 3 weeks in 84% patients. Serum creatinine values returned to normal within one week in 11 (44%), while in 10 (40%) patients it returned in 3-week time. Only one patient (4%) took a longer time (5 weeks) to attain the baseline levels. One female patient aged 49 years, with baseline serum creatinine of 1.2 mg/dL, had received 40 mL of 300 mg% of iohexol (12 g iodine) for a CECT head study. She developed acute renal failure and had to undergo dialysis. Another 55-year-old male patient with suspected renal cell carcinoma (pre-CT serum creatinine of 1.6 mg/dL) received 100 mL of 240 mg% of iohexol (24 g iodine), developed CIN (postprocedure serum creatinine 2.06 mg/dL), and died at home, and the cause of death could not be ascertained. Both these patients had mild dehydration and chronic kidney disease. One patient was lost to follow-up.

## 5. Discussion

This observational study was conducted in a hospital based radiology set-up which entertains both routine and emergency CT and IVU requisitions requiring intravenous CM administration. 250 patients constituted the final study group for determining the incidence of CIN and to identify various patients and contrast related risk factors.

As a routine practice, the baseline serum creatinine levels (done within a week for indoor patients) were estimated to screen patients with decreased eGFR calculated using MDRD formula before administering intravascular iodinated CM. Serum creatinine of 1.8 mg/dL corresponding to an eGFR of 45 mL/min/1.73 m^2^ has been found to be significant for the development of AKI in patients receiving CM in comparison to the patients not receiving any contrast [24]. In our study sample, eGFR of less than 45 mL/min/1.73 m^2^ was used as an exclusion criterion and therefore patients did not carry any additional risk of developing CIN nor required any preimaging (CECT or IVU) hydration therapy. Thus, low risk patients not requiring any preventive therapy before administration of IV contrast were recruited for the study.

The preprocedural mean serum creatinine value of 0.905 ± 0.248 mg/dL was compared with the postprocedural mean serum creatinine value of 0.966 ± 0.300 mg/dL in the study sample. The postinvestigation increase in the serum creatinine levels was found to be statistically significant, suggesting a significant deterioration in renal function. Previous studies indicated a definite pathophysiological renal insult occurring due to administration of intravascular iodinated contrast because of an increased viscosity of blood leading to sluggish blood flow which induces local hypoxia [25–27]. Besides reducing the renal perfusion, it also causes direct toxicity to tubular cells resulting in deterioration of the renal function [25, 28].

Of the 250 patients, 25 patients satisfied the selected criteria for CIN, resulting in an incidence of 10% following intravenous contrast administration. This incidence of 10% is alarming in low risk (patients with eGFR of 45 mL/min/1.73 m^2^ or more) patients sample receiving contrast through the intravenous route which has been considered to be safe in most past studies [29]. However, in a recent study, incidence of AKI was calculated following noncontrast and contrast CT and concluded that it is inversely proportional to baseline eGFR and varies from 1.2% to 14% [30]. The incidence was not statistically different to a similar study conducted by Mitchell et al. on 633 patients undergoing emergency CECT examination (*p* > 0.05) [8]. On the contrary, our patients underwent routine IVU or CECT study. A varying incidence of CIN is present in the literature, ranging from 1.3 to 14.5% [4]. This wide range might be due to different criteria used for diagnosing CIN, wide variation in the study sample, and administration of intravascular contrast through varying routes. There is no consensus regarding the clinical significance of a mild but statistically significant increase in the serum creatinine following contrast administration. Hayman advocated that a change of 0.3 mg/dL of serum creatinine [31] has no clinical significance, whereas Levy et al. found that even a small increase in serum creatinine increases mortality by causing a significant decrease in GFR and thus the renal function [32].

In the CIN group of 25 patients, serum creatinine reached the baseline level within a week in a maximum of 44% patients, while in 40% baseline levels were obtained in up to 3-week time. One patient (4%) took longer to recover and one patient died due to unknown cause. One patient (4%) with known kidney disease (with preinvestigation serum creatinine of 1.2 mg/dL) developed acute renal failure requiring dialysis. Thus, most (84%) of the patients developing CIN had a full recovery in 3-week time postinvestigation. This reiterates the previous study which states that CIN is a transient process with serum creatinine levels rising within 24 hours of contrast administration, peaks within 3–5 days to return to the baseline levels within two weeks [7]. Some investigators believe that there is no biological significance of CIN whereas few are still unclear regarding its clinical significance. The CIN consensus working panel has found CIN to be responsible for 11% of all cases of kidney impairment requiring hospitalization, but with intra-arterial use of CM [33]. Some authors have estimated the incidence of renal failure to be less than 2% [34–36]. With intravenous contrast, Thomsen and Morcos [5] also found significant increase of mortality or renal failure in CIN patients. Our study did not focus on this aspect and hence the significance of mortality/morbidity due to CIN cannot be commented upon. But as the incidence of CIN is quite high for a routine setting as reflected by our work, it further requires an intensive study to study its clinical implications.

The study sample was divided into two groups: “CIN” and “No CIN” groups to see whether the distribution of the risk factors was significantly different in the two groups or not.

Demographic variables like age and weight were homogenous in both CIN and No CIN groups indicating no role in the occurrence of CIN. Sex ratio was also statistically similar in both the groups. Similar observation of no influence of gender was made by Evola et al. in their study [37]. Though the mean age was higher in “CIN group” as compared to “No CIN group,” it was not found to be statistically significant (*p* > 0.05) in our study, whereas older age is considered to be an independent predictor of CIN [37–39]. Our results can be explained by the relatively younger population of mean age 41.41 ± 16.63 years constituting the study group in comparison to 65.32 ± 12.02 years for others [37]. Mean weight had no correlation to the occurrence of CIN and no reference exists in the literature.

Multiple factors have been identified for development of CIN, with reduction in renal function being the most significant, though not studied specifically in the past studies. Traditional risk factors of CIN like dehydration, preexisting renal disease, heart failure, and history of previous contrast administration were found to be significant in the development of CIN with a high relative risk. Dehydration increases the risk of developing CIN due to decreased intravascular volume resulting in decreased renal blood flow and ischemia and thus, exaggerating the renal insult [40, 41].

Preexisting renal disease is an independent risk factor of nephrotoxicity and development of CIN. It is the single greatest risk factor with the severity of CIN increasing in proportion to the baseline renal insufficiency [42]. The higher is the baseline serum creatinine value and the greater is the risk [29, 43], but not in patients with mild decrease in renal function (eGFR > 45 and < 90 mL/min/1.73 m^2^), not requiring prophylactic therapy.

The mean preprocedural serum creatinine was 0.912 ± 0.266 mg/dL in the “CIN” and 0.904 ± 0.246 mg/dL in the “NO CIN” group, corresponding to mean eGFR of 85 (68–108.5) mL/min/1.73 m^2^ and 88 (70.5–107.5) mL/min/1.73 m^2^, respectively. This difference was not found to be significant in our study. Despite all patients having eGFR > 45 mL/min/1.73 m^2^ (low risk patients), still incidence of CIN was quite high (10%), thus emphasizing the need for detection of risk factors which may independently or in conjunction with mild renal derangement cause significant renal deterioration and development of CIN.

There was a significant increase in the risk of CIN in patients of cardiac failure in our study. Previous researchers found that reduction in effective intravascular volume associated with reduced cardiac output decreases the renal perfusion and there is an increased risk of CIN [13, 40] as also found in our study.

Two doses given 24 to 48 hours apart increase the risk of CIN [44, 45]. But CIN was also found in patients who had received previous (within two weeks) intravenous contrast in our study raising the possibility of significant renal derangement for up to two weeks after injecting contrast, which may have been responsible for the finding in our study.

Past renal surgery, diabetes mellitus, and hypertension are found to have high relative risks but not significantly associated with the occurrence of CIN in the present study. Though an increased incidence of CIN is seen in diabetic patients in our study, comparison between the two groups failed to reach any statistical significance (*p* > 0.05), hence not being identified as a separate risk factor in our study, in contradiction to the available literature [11]. Hypertensive patients were found to have no statistical difference showing no correlation to development of CIN in our study sample. Most of the published studies and reviews did not find arterial blood pressure as a separate risk factor [31, 37, 46] although it is included in suggested list of risk factors in 2013 ACR Manual on Contrast Media [47].

The previous renal surgery was not qualified as a separate risk factor in our study as also in most studies and meta-analyses [26, 36]. Nephrotoxic drug intake and abnormal hematological findings are not related to the development of CIN and are not yet established as risk factors in existing literature.

Contrast of different types based on their physicochemical properties has conflicting reports, regarding its relation to the occurrence of CIN. Contrast related factors like ionicity, structure, and osmolality are not related to development of CIN with comparable incidence in ionic (7.9%) and nonionic (10.7%) CM groups in our study as well (*p* = 0.63). A meta-analysis contradicts our study by reporting significantly reduced incidence of CIN with use of “low osmolar” compared to “ionic high osmolar” CM, though in patients with preexisting CKD [48]. Volume of contrast administered intravenously was directly linked to the occurrence of CIN in the present study as already established [37, 49]. The total iodine received by patients may have a possible relation to the development of CIN as suggested by our study, with *p* value of 0.05 reaching the limit of significance. This could be likely due to the direct relation of total iodine content with the volume of CM. No literature is available in this context opening up future prospects for the same. This could prove to be significant in CT-angiographic and CT-perfusion studies requiring lower volume but a higher strength of contrast and thus higher iodine content.

Dehydration, preexisting renal disease, cardiac failure, previous contrast administration, and volume of contrast were identified as significant risk factors for the development of CIN in our study. These factors are quite similar to the risk factors identified by Mehran and Nikolsky [36]; however, diabetes, advanced age, and baseline renal function were not found to be significant in our study. This possibly occurred due to a relatively younger study population, exclusion of patients with poor baseline renal function, and limited sample size.

The statistically significant rise in serum creatinine levels after imaging requiring IV contrast successfully established the occurrence of a definite renal insult with incidence of CIN being 10%. The study design was able to recognize the important risk factors in the study and also suggest their relative risk of developing CIN.

Limitations of the study were that no control group was present. A relatively smaller sample size though statistically adequate resulted in inadequate number to study significance of few already known risk factors (like previous renal surgery, diabetes mellitus, and hypertension) for development of CIN. Only the “CIN” and not the “NO CIN” group of patients were followed up after the contrast procedure. Long term complications in patients developing CIN needs to be studied. Also, risk associated with presence of multiple risk factors needs to be investigated.

## 6. Conclusions

Incidence of CIN is 10% in patients undergoing CECT or IVU examinations with IV iodinated CM. However, no relationship could be established between the occurrence of CIN and the base line renal functions. There is a definite renal insult with IV iodinated CM showing significant increase in postinvestigation serum creatinine. CIN is transient in majority with recovery occurring in 84% patients within 3 weeks. Traditional risk factors of CIN including dehydration, preexisting renal disease, cardiac failure, and previous intravascular contrast administration are related to the development of CIN with a high relative risk. Previous renal surgery, diabetes mellitus, and hypertension have high relative risks but are not significantly associated with the occurrence of CIN. Volume of IV contrast is directly linked to occurrence of CIN. CIN is an important concern for radiologists with a high incidence in our set-up. The radiologists are advised to recognize this intrinsic risk to iodinated contrast media, identifying the patients at risk of developing CIN and those requiring hospital care.

Based on the study, a performa was developed to be used for IV administration of CM to identify “at risk patients” for developing CIN with routine use of intravenous contrast, detect CIN, and follow up these patients (Table 4). We suggest all the fellow radiologists to utilize this performa to address the sensitive issue of contrast induced nephropathy.

## Figures and Tables

**Table 1:** Demographic distribution between “CIN group” and “No CIN group.”

	CIN (*n* = 25)	No CIN (*n* = 225)	*p* value
Females	9 (36%)	94 (41.8%)	0.671
Males	16 (64%)	131 (58.2%)	
Age (years)	45.12 ± 17.82	41.00 ± 16.48	0.241
Weight (kg)	58.44 ± 13.00	55.00 ± 10.12	0.119

^*∗*^ *p* value calculated using chi-square test, correlation to be significant if *p* < 0.05.

**Table 2:** Distribution of clinical risk factors between “CIN” group and “No CIN” group.

Risk factors	CIN (*n* = 25)	No CIN (*n* = 225)	*p* value	Relative risks	95% CI	
					Lower	Upper
Dehydration	8 (32%)	20 (8.9%)	0.003	3.73	1.78	7.84
Abnormal routine blood investigations	7 (29%)	48 (21.8%)	0.457	1.35	0.59	3.06
Preexisting renal disease	6 (24%)	13 (5.8%)	0.006	3.84	1.74	8.45
Diabetes mellitus	6 (24%)	25 (11.1%)	0.100	2.23	0.97	5.15
Previous contrast use	6 (24%)	2 (0.9%)	0.000	9.56	5.30	17.21
Hypertension	4 (16%)	17 (7.6%)	0.143	2.08	0.79	5.49
Previous renal surgery	2 (8%)	5 (2.2%)	0.148	3.02	0.88	10.37
Cardiac failure	2 (8%)	1 (0.4%)	0.027	7.16	2.94	17.43
Nephrotoxic drug intake	3 (12%)	19 (8.4%)	0.469	1.41	0.46	4.35

^*∗*^ *p* value calculated using Fisher's exact test or chi-square test wherever appropriate; correlation was considered significant if *p* < 0.05.

**Table 3:** Contrast characteristics in “CIN” and “No CIN” groups.

Contrast characteristics		CIN (*n* = 25)	No CIN (*n* = 225)	*p* value
Ionicity	Ionic	5 (20%)	58 (25.8%)	0.633
	Nonionic	20 (80%)	167 (74.2%)	

Osmolarity	HOCM	4 (16%)	43 (19.11%)	1.000
	LOCM	21 (84%)	182 (80.89%)	

Structure	Dimer	1 (4%)	15 (6.67%)	1.000
	Monomer	24 (96%)	210 (93.33%)	

Total iodine (g)		22.37 ± 5.312	20.21 ± 5.30	0.05

Volume (mL)		73.20 ± 18.19	65.11 ± 17.93	0.03

**Table 4:** Recommended requisition form for contrast enhanced investigations in our department.

Name	Consent^*∗*^		
Age	Baseline ( not more than 1 week old) serum creatinine (mg/dL) = eGFR calculated (mL/min/1.73 m^2^) = If < 45 mL/min/1.73 m^2^ defer CECT/IVP, take preventive measures		

Sex	H/o contrast allergy, drug allergy, or allergic condition; if yes, defer Ix and preventive measures taken		

Weight	(1) Preexisting renal disease	Y/N	Type of disease

Clinical indication for IVP/CECT	(2) Dehydration on history or clinical exam	Y/N	

Ix required IVP/CECT study and ID	(3) H/o previous contrast (within 2 wks)	Y/N	IV or IA, type of CM

Any significant past or present medical illness	(4) H/o heart failure	Y/N	Past/present
	(5) H/o renal surgery	Y/N	Type of surgery

Hb/TLC/CRP	(6) H/o diabetes mellitus	Y/N	Recent fasting blood sugar level
	(7) H/o hypertension	Y/N	Blood pressure = mm of Hg
	(8) H/o nephrotoxic drug intake	Y/N	Type of drug

If one of the risk factors 1–4 or two of risk factors 5–8 or subnormal renal function (eGFR 46 to 90 mL/min/1.73 m^2^) or if volume of administered IV contrast is equal to or more than 100 mL			

Repeat S creatinine after 2-3 days of Ix, postprocedural S. Creatinine level =			

Postprocedural S. Creatinine raised Yes/No % increase =			

If increase > 25% or is by an absolute value of 0.5 mg/dL, CIN is diagnosed; Group allotted: CIN or No CIN			

If CIN present, serum creatinine is repeated weekly and refer to nephrologist if there is clinical deterioration			

If not, send back to referring clinician			

^*∗*^Consent to be taken on separate form, Ix: investigation.
